# Puberty timing and adiposity change across childhood and adolescence: disentangling cause and consequence

**DOI:** 10.1093/humrep/deaa213

**Published:** 2020-11-26

**Authors:** Linda M O’Keeffe, Monika Frysz, Joshua A Bell, Laura D Howe, Abigail Fraser

**Affiliations:** 1 School of Public Health, University College Cork, Cork, Ireland; 2 MRC Integrative Epidemiology Unit at the University of Bristol, Bristol, UK; 3 Population Health Sciences, Bristol Medical School, Bristol, UK

**Keywords:** puberty, adiposity, prevention, life course, epidemiology / ALSPAC

## Abstract

**STUDY QUESTION:**

Is earlier puberty more likely a result of adiposity gain in childhood than a cause of adiposity gain in adulthood?

**SUMMARY ANSWER:**

Pre-pubertal fat mass is associated with earlier puberty timing but puberty timing is not associated with post-pubertal fat mass change.

**WHAT IS KNOWN ALREADY:**

Age at puberty onset has decreased substantially in the last several decades. Whether reducing childhood adiposity prevents earlier puberty and if early puberty prevention itself also has additional independent benefits for prevention of adult adiposity is not well understood.

**STUDY DESIGN, SIZE, DURATION:**

Prospective birth cohort study of 4176 participants born in 1991/1992 with 18 232 repeated measures of fat mass from age 9 to 18 years.

**PARTICIPANTS/MATERIALS, SETTING, METHODS:**

We used repeated measures of height from 5 to 20 years to identify puberty timing (age at peak height velocity, aPHV) and repeated measures of directly measured fat mass from age 9 to 18 years, from a contemporary UK birth cohort study to model fat mass trajectories by chronological age and by time before and after puberty onset. We then examined associations of these trajectories with puberty timing separately in females and males.

**MAIN RESULTS AND THE ROLE OF CHANCE:**

In models by chronological age, a 1-year later aPHV was associated with 20.5% (95% confidence interval (CI): 18.6–22.4%) and 23.4% (95% (CI): 21.3–25.5%) lower fat mass in females and males, respectively, at 9 years. These differences were smaller at age 18 years: 7.8% (95% (CI): 5.9–9.6%) and 12.4% (95% (CI): 9.6–15.2%) lower fat mass in females and males per year later aPHV. Trajectories of fat mass by time before and after puberty provided strong evidence for an association of pre-pubertal fat mass with puberty timing, and little evidence of an association of puberty timing with post-pubertal fat mass change. The role of chance is likely to be small in this study given the large sample sizes available.

**LIMITATIONS, REASONS FOR CAUTION:**

Participants included in our analyses were more socially advantaged than those excluded. The findings of this work may not apply to non-White populations and further work examining associations of puberty timing and fat mass in other ethnicities is required.

**WIDER IMPLICATIONS OF THE FINDINGS:**

Previous research has relied on self-reported measures of puberty timing such as age of voice breaking in males, has lacked data on pre-and post-pubertal adiposity together and relied predominantly on indirect measures of adiposity such as BMI. This has led to conflicting results on the nature and direction of the association between puberty timing and adiposity in females and males. Our work provides important clarity on this, suggesting that prevention of adiposity in childhood is key for prevention of early puberty, adult adiposity and associated cardiovascular risk. In contrast, our findings suggest that prevention of early puberty without prevention of childhood adiposity would have little impact on prevention of adult adiposity.

**STUDY FUNDING/COMPETING INTEREST(S):**

The UK Medical Research Council and Wellcome (Grant ref: 102215/2/13/2) and the University of Bristol provide core support for Avon Longitudinal Study of Parents and Children (ALSPAC). L.M.O.K. is supported by a UK Medical Research Council Population Health Scientist fellowship (MR/M014509/1) and a Health Research Board (HRB) of Ireland Emerging Investigator Award (EIA-FA-2019-007 SCaRLeT). J.A.B. is supported by the Elizabeth Blackwell Institute for Health Research, University of Bristol and the Wellcome Trust Institutional Strategic Support Fund (204813/Z/16/Z). L.D.H. and A.F. are supported by Career Development Awards from the UK Medical Research Council (grants MR/M020894/1 and MR/M009351/1, respectively). All authors work in a unit that receives funds from the UK Medical Research Council (grant MC_UU_00011/3, MC_UU_00011/6). No competing interests to declare.

**TRIAL REGISTRATION NUMBER:**

N/A.

## Introduction

Age at puberty onset has decreased substantially among females since the mid-1900s (Euling *et al.*, 2008). Secular trends in males are less well understood due to imprecise markers of pubertal age such as age at voice breaking compared with age at menarche among females (Euling *et al.*, 2008; Kaplowitz, 2008). Earlier puberty directly results in younger fertility and thus carries important social implications (Golub *et al.*, 2008), but it may also have adverse implications for health, with evidence of increased risk of adult obesity, type 2 diabetes, cardiovascular disease and several cancers in both sexes (Prentice and Viner, 2013; Canoy *et al.*, 2014; Charalampopoulos *et al.*, 2014; Day *et al.*, 2015).

Higher BMI before puberty onset is associated with earlier menarche in females, raising the possibility that much of the associations of puberty timing with health in later life reflects tracking of adiposity from childhood (Wang, 2002; Lee *et al.*, 2007; Buyken *et al.*, 2008; Kaplowitz, 2008; Kivimèki *et al.*, 2008; Silventoinen *et al.*, 2008). In males, however, some studies have found that higher childhood BMI is associated with later puberty (Wang, 2002; Lee et al., 2010; 2016), while others have found associations similar to those observed in females (He and Karlberg, 2001; Sandhu *et al.*, 2006; Buyken *et al.*, 2008; Silventoinen *et al.*, 2008; Aksglaede *et al.*, 2009). In a systematic review and meta-analysis of 11 cohort studies, pre-pubertal obesity among females was associated with earlier menarche but there was insufficient and inconsistent evidence in males (Li *et al.*, 2017). A recent Mendelian randomisation (MR) analysis suggested that earlier age at menarche caused higher adult BMI but lacked data on pre-pubertal BMI for adjustment (Gill *et al.*, 2018). In contrast, another recent MR which did have pre-pubertal BMI data suggested that associations of earlier puberty with higher adult BMI were largely confounded by childhood BMI (Bell *et al.*, 2018). Thus, whether pre-pubertal adiposity plays a role in early puberty in females and males and if early puberty also has additional independent associations with post-pubertal adiposity is unclear.

Most prospective studies to date have used self-reported measures of puberty timing such as age of voice breaking in males, have lacked data on pre- and post-pubertal adiposity together and relied predominantly on indirect measures of adiposity such as BMI. In addition, disentangling direction of causality of puberty timing and adiposity may be difficult in available MR studies due to a shared genetic architecture between adiposity and puberty timing (age at menarche) (Day *et al.*, 2015). In this study, we aimed to better understand the association between puberty timing and pre- and post-pubertal adiposity change by examining an objective growth-based measure of pubertal onset (age at peak height velocity (aPHV)) in relation to change in directly measured dual-energy X-ray absorptiometry-derived (DXA-derived) fat mass across childhood and adolescence.

## Materials and methods

### Study participants

Data were from the Avon Longitudinal Study of Parents and Children (ALSPAC), a prospective birth cohort study in southwest England (Boyd *et al.*, 2013; Fraser *et al.*, 2013). Pregnant women resident in one of the three Bristol-based health districts with an expected delivery date between 1 April 1991 and 31 December 1992 were invited to participate. The study is described elsewhere in detail (Boyd *et al.*, 2013; Fraser *et al.*, 2013). ALSPAC initially enrolled a cohort of 14 451 pregnancies, from which 13 867 live births occurred in 13 761 women. Follow-up has included parent- and child-completed questionnaires, clinic attendance and links to routine data. Research clinics were held when the participants were ∼7, 9, 10, 11, 13, 15 and 18 years.

### Ethical approval

Ethical approval for the study was obtained from the ALSPAC Ethics and Law Committee and the Local Research Ethics Committees. The study website contains details of all the data that are available through a fully searchable data dictionary http://www.bristol.ac.uk/alspac/researchers/our-data/ (University of Bristol, 2020).

### Data

#### Assessment of puberty timing

Puberty is a period of intense hormonal activity and rapid growth, of which the most striking feature is the spurt in height (Cole *et al.*, 2014). aPHV is a validated measure of pubertal timing (Cole *et al.*, 2014) captured using Superimposition by Translation and Rotation (SITAR), a non-linear multilevel model with natural cubic splines which estimates the population average growth curve and departures from it as random effects (Cole *et al.*, 2010; Simpkin *et al.*, 2017). Using SITAR, PHV was identified in ALSPAC participants using numerical differentiation of the individually predicted growth curves, with aPHV being the age at which the maximum velocity is observed (Cole *et al.*, 2010; Simpkin *et al.*, 2017; Frysz *et al.*, 2018). Repeated height data included measurements from research clinics. Individuals with at least one measurement of height in each of the age ranges 5 to <10, 10 to <15 and 15–20 years are included here. Data were analysed for females and males separately. The model was fitted using the SITAR package in R version 3.4.1, as described elsewhere (Frysz *et al.*, 2018). Further details on how aPHV was derived are described elsewhere (Frysz *et al.*, 2018) and in Supplementary Methods, Table SI and Fig. S1 of Supplementary Material.

#### Assessment of adiposity

Adiposity was assessed via total body fat mass (in kilogram, less head) as derived from whole-body DXA scans performed five times at ages 9, 11, 13, 15 and 18 years using a GE Lunar Prodigy (Madison, WI, USA) narrow fan-beam densitometer.

#### Confounders

We considered the following as potential confounders in our analysis: birth weight, gestational age, maternal education, parity, maternal smoking during pregnancy, maternal age, maternal pre-pregnancy BMI, household social class, marital status, partner education and ever breastfeeding all measured by mother-or mother’s partner-completed questionnaires; details in Supplementary Methods). The distribution of confounders included in our analyses, including the proportion of missing data for each confounder by fourths of aPHV, is also shown in Supplementary Table SII; note the table demonstrates minor differences in the proportion of missing confounder data by fourths of aPHV.

### Statistical analysis

Multilevel models were used to examine change in fat mass during childhood and adolescence (Laird and Ware, 1982; Goldstein, 1995). Using terms such as polynomials and splines to account for non-linearity in the trajectory, such models can estimate mean trajectories of the outcome while accounting for the non-independence or clustering of repeated measurements within individuals, change in scale and variance of measures over time, and differences in the number and timing of measurements between individuals (using all available data from all eligible participants under a missing at-random assumption) (Howe *et al.*, 2013; Tilling *et al.*, 2014). Participants that reported being pregnant at the 18-year clinic were excluded from the multilevel models at that time point only (N = 6). Participants that had a measure of aPHV, at least one measure of fat mass from 9 to 18 years and complete data on all confounders were included in analyses, leading to a total sample of 4176 (2186 females and 1990 males).

All analyses were performed separately for females and males. aPHV was normally distributed in both sexes. Linearity of associations of aPHV with fat mass was examined by comparing the model fit of regressions of fat mass on aPHV, with continuous aPHV and fourths of aPHV examined as continuous exposures. Model fit was then formally tested using a likelihood ratio test. Prior to analysis, aPHV was centred on the sex-specific mean of aPHV for females and males. Fat mass was also log transformed due to its skewed distribution. All models were adjusted for height using the time- and sex-varying power of height that best resulted in a height-invariant measure, described in detail elsewhere (O'Keeffe *et al.*, 2019a,b). We performed unadjusted and confounder-adjusted analyses on participants with complete data (N = 4176) for all models.

From all models, we back-transformed the difference in fat mass trajectories per year of aPHV and the average trajectory for the 10th, median and 90th sex-specific percentile of aPHV. The back-transformed difference in fat mass per year of aPHV is a ratio of geometric means, expressed here as a percentage difference per year of aPHV. The average trajectories back-transformed from the log scale are in original units (kg) and are presented in figures.

#### Models for fat mass trajectories

A common approach to modelling change over time using multilevel models involves examining change by chronological age (O'Keeffe *et al.*, 2018a,b, 2020). However, when change before or after a specified event is of interest (e.g. onset of puberty or menopause), it is also possible to model change according to other time metrics such as time before and/or after the event. Thus, to gain a greater understanding of the association of aPHV with change in fat mass during childhood and adolescence, we modelled trajectories of fat mass in two ways: by chronological age, and separately by time before and after puberty.

#### Model 1: Chronological age-based models

Fat mass was previously modelled according to chronological age using linear spine multilevel models, with three periods of linear change (9 to <13, 13 to <15 and 15–18 years) (O'Keeffe *et al.*, 2018a,b, 2019a,b). Thus, for this analysis, we examined whether this model was appropriate for modelling change over time within quartiles of pubertal age to ensure that model fit was adequate across the entire distribution of pubertal age. Subsequently, the association between aPHV and chronological age-based trajectories was then examined for females and males by including an interaction between centred sex-specific aPHV and the intercept (age 9 years) and each spline period, providing an estimate of the difference in the average trajectory of fat mass from age 9 to 18 years, per year later aPHV. Confounders were included as interactions with both the intercept and linear slopes; note, inclusion of interaction terms between variables (exposures/confounders) and the outcome trajectory is the standard approach to examining associations of an exposure with an outcome trajectory and adjusting this outcome trajectory for confounders in multilevel models (Howe *et al.*, 2013; Tilling *et al.*, 2014). A main effect for the exposure/confounder plus interaction terms between exposures/confounders and linear spline terms ensures that the effect of the exposure/confounder on the intercept (here, the value of fat mass at 9 years) and each linear spline term is modelled. For exposures (here aPHV), this allows us to estimate differences in trajectories of fat mass from 9 to 18 years by different values of aPHV. For confounders, this ensures that the full trajectory of fat mass from 9 to 18 years is adjusted for the confounders of interest.

#### Model 2: Pubertal age-based models

The purpose of the pubertal age-based model was to examine whether changes in fat mass before or after puberty onset differ by aPHV. In order to select an appropriate model, we examined observed data for fat mass in females and males by sex-specific quartiles of aPHV. Based on the observed data, a selection of suitable models was examined, each with different numbers of pre- and post-pubertal change periods. We compared observed and predicted values of fat mass for these models by sex-specific quartiles of pubertal age to examine model fit. In females, the final model selected for fat mass included two periods of change (pre-puberty and post-puberty). The final model for fat mass in males had three periods of change (from age 9 to 3 years before puberty, from 3 years before puberty to puberty (i.e. aPHV) and from puberty to the end of follow-up at 18 years). Differences in the rate of change in fat mass before and after puberty by aPHV were then modelled by including an interaction between centred sex-specific aPHV and the intercept (fat mass at puberty) and each linear spline period (one pre- and post-pubertal spline period for females and two pre- and one post-pubertal spline period for males). This model provided insight into whether different ages at PHV were accompanied by different rates of change in fat mass before and after puberty. Confounders were included as interactions with the intercept and linear slopes, as described above for chronological age-based models. All trajectories were modelled in MLwiN version 3.04 (University of Bristol Centre for Multilevel Modelling, 2019), called from Stata version 16 (StataCorp, 2019) using the runmlwin command (University of Bristol Centre for Multilevel Modelling, 2016).

Further details on model selection are included in Supplementary Methods and details of model fit for both models are included in Supplementary Tables SIII and SIV.

#### Additional and sensitivity analyses

We examined the characteristics of mothers of participants included in our analysis compared with mothers of participants excluded from our analysis due to missing exposure, outcome or confounder data to better understand generalisability and the potential for selection bias. We performed unadjusted analyses on the sample of participants that had data on aPHV and at least one measure of fat mass from 9 to 18 years; this analysis included an additional 1517 participants excluded from our main analysis due to missing confounder data (total N = 5693). We regressed observed fat mass at 9 years (first available measure) and 18 years (last occasion of measurement) on aPHV in females and males and compared results to those obtained from the multilevel models at these ages. We performed sensitivity analyses restricting the sample to participants with at least one fat mass measure before and one after aPHV to examine whether results from the main analysis were driven by participants with only a single pre- or post-puberty fat mass measure. We examined whether the association of self-reported age at menarche with fat mass during childhood and adolescence was similar to findings for the association of aPHV and fat mass among females. In addition, we examined whether our findings were similar in analyses when non-White ALSPAC participants were excluded from analyses (N = 178 excluded).

## Results

The characteristics of participants included in analyses (N = 4176), by sex, are shown in Table I. Mean aPHV was 11.7 (standard deviation (SD) = 0.8) for females (N = 2186) and 13.6 (SD = 0.9) for males (N = 1990). Findings from linearity tests of aPHV and fat mass at each age demonstrated little evidence of departures from linearity, allowing aPHV to be examined as a continuous exposure (Supplementary Table SV). Mothers of participants included in the analysis were more likely to be married, have higher household social class, higher education, higher partner education, lower prevalence of smoking during pregnancy, lower parity and higher maternal age compared with participants excluded due to missing exposure, outcome or confounder data (Supplementary Table SVI). Participants included in analyses were more likely to be female and higher birth weight compared with those excluded. However, gestational age at birth, maternal BMI, aPHV and fat mass at most occasions were similar between included and excluded participants (Supplementary Table SVI).

**Table I:** Characteristics of ALSPAC participants included in the analysis, by sex.

	**Females** **N = 2186**	**Males** **N = 1990**
	n (%)	n (%)
Maternal marital status		
Never married	269 (12.3)	196 (9.8)
Widowed	<5*	<5*
Divorced	65 (3.0)	70 (3.5)
Separated	20 (0.9)	15 (0.8)
1st marriage	1701 (77.8)	1564 (78.6)
Marriage 2 or 3	130 (5.9)	142 (7.1)
Household social class^†^		
Professional	377 (17.2)	390 (19.6)
Managerial & Technical	1009 (46.2)	947 (47.6)
Non-Manual	526 (24.1)	449 (22.6)
Manual	198 (9.1)	142 (7.1)
Part Skilled & Unskilled	76 (3.5)	62 (3.1)
Maternal education		
Less than O level	360 (16.5)	311 (15.6)
O level	781 (35.7)	694 (34.9)
A level	621 (28.4)	602 (30.3)
Degree or above	424 (19.4)	383 (19.2)
Mother’s Partner’s highest educational qualification		
Less than O level	553 (25.3)	426 (21.4)
O level	471 (21.5)	444 (22.3)
A level	640 (29.3)	583 (29.3)
Degree or Above	522 (23.9)	537 (27.0)
Maternal smoking during pregnancy		
No	1874 (85.7)	1707 (85.8)
Yes	312 (14.3)	283 (14.2)
Parity		
0	1068 (48.9)	981 (49.3)
1	796 (36.4)	684 (34.4)
2	322 (14.7)	325 (16.3)
Breastfeeding		
Exclusive	841 (38.5)	697 (35.0)
Non-exclusive	1006 (46.0)	1034 (52.0)
Never	339 (15.5)	259 (13.0)

	**Mean (SD)**	**Mean (SD)**

Gestational age (weeks)	39.6 (1.6)	39.4 (1.9)
Birth weight (g)	3393.0 (483.6)	3481.4 (574.5)
Maternal pre-pregnancy BMI (kg/m^2^)	22.8 (3.6)	22.9 (3.7)
Maternal age at delivery (years)	29.3 (4.3)	29.7 (4.4)

* Exact numbers and percentages not shown due to potential for disclosure.

† Household social class was measured as the highest of the mother’s or her partner’s occupational social class using data on job title and details of occupation collected about the mother and her partner from the mother’s questionnaire at 32 weeks of gestation. Social class was derived using the standard occupational classification (SOC) codes developed by the United Kingdom Office of Population Census and Surveys and classified as I professional, II managerial and technical, IIINM non-manual, IIIM manual, and IV and V part skilled occupations and unskilled occupations.

SD, standard deviation.

### Puberty timing and adiposity change from models by chronological age

**Figure 1.**
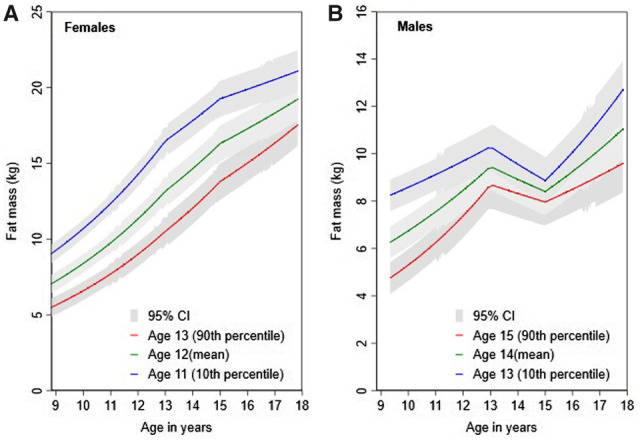
**Mean trajectories of height-adjusted fat mass in females and males from 9 to 18 years for the 10th, median and 90th sex-specific percentiles of age at peak height velocity from multilevel models based on chronological age.** Ages presented are rounded for ease of interpretation. Exact ages are 12.9, 11.7 and 10.7 years for females and 14.7, 13.6 and 12.5 years for males. Age at peak height velocity is normally distributed and median is equal to mean. Models are adjusted for birth weight, gestational age, maternal education, parity, maternal smoking during pregnancy, maternal age, maternal pre-pregnancy BMI, household social class, marital status, partner education and breastfeeding. CI, confidence interval.

A 1-year later aPHV in females was associated with a lower fat mass at 9 years (Table II) and faster gain in fat mass from 9 to 18 years. By 18 years, the mean difference per year later aPHV persisted but was smaller. In males, associations were comparable; a 1-year later aPHV was associated with lower fat mass at 9 years which reduced to a smaller difference at age 18 (Table II). Mean-adjusted trajectories of fat mass from 9 to 18 years for the 10th (age 11 in females and age 13 males), 50th (age 12 in females and age 14 in males) and 90th (age 13 in females and age 15 in males) sex-specific percentiles of aPHV by chronological age are presented in Fig. 1.

**Table II:** Adjusted mean trajectory and mean difference in trajectory of height-adjusted fat mass per year later age at peak height velocity, from chronological age multilevel models.

	Mean trajectory (95% CI) of height-adjusted fat mass		**Mean difference in height-adjusted fat mass (95% CI)** **per year later age at peak height velocity**	
**Females**	Age 9y (kg)^*^	7.23 (6.43, 8.02)	Age 9y (% difference)^‡^	−20.49 (−22.38, −18.59)
	9 - <13y (% /y)^†^	16.49 (13.83, 19.16)	9 - <13y (% difference /y)^§^	0.67 (0.19, 1.15)
	13 - <15y (% /y)^†^	10.18 (6.00, 14.36)	13 - <15y (% difference /y)^§^	2.54 (1.71, 3.37)
	15 - 18y (% /y)^†^	8.09 (5.36, 10.82)	15 - 18y (% difference /y)^§^	2.42 (1.86, 2.98)
	Age 18y (kg)^*^	20.40 (18.50, 22.30)	Age 18y (% difference)^‡^	−7.76 (−9.62, −5.89)

**Males**	Age 9y (kg)^*^	6.03 (5.26, 6.80)	Age 9y (% difference)^‡^	−23.37 (−25.47, −21.28)
	9 - <13y (% /y)^†^	11.93 (10.72, 13.14)	9 - <13y (% difference /y)^§^	4.85 (4.24, 5.46)
	13 - <15y (%/y)^†^	−5.77 (−6.82, −4.72)	13 - <15y (% difference /y)^§^	1.38 (0.31, 2.46)
	15 - 18y (% /y)^†^	10.09 (9.25, 10.94)	15 - 18y (% difference /y)^§^	−2.73 (−3.56, −1.89)
	Age 18y (kg)^*^	11.21 (9.80, 12.63)	Age 18y (% difference)^‡^	−12.38 (−15.19, −9.57)

Association of age at peak height velocity (per year later) with fat mass at 9 years and change in fat mass 9–18 years in females and males separately. Mean trajectory is centred on the sex-specific mean of age at peak height velocity for each sex (age ∼11.7 for females and age ∼13.6 for males). The difference in fat mass per year of age at peak height velocity is back-transformed from the log scale for ease of interpretation and is a ratio of geometric means, expressed as a percentage difference. Adjusted for birth weight, gestational age, maternal education, parity, maternal smoking during pregnancy, maternal age, maternal pre-pregnancy BMI, household social class, marital status, partner education and breastfeeding.

* Mean height-adjusted fat mass at 9 and 18 in kilograms.

† Percentage change per year in height-adjusted fat mass.

‡ Percentage difference in fat mass at 9 and 18 years per year later age at peak height velocity.

§ Percentage difference in change per year, per year later age at peak height velocity.

CI, confidence interval.

**Table III:** Adjusted mean trajectory and mean difference in trajectory of height-adjusted fat mass per year later age at peak height velocity, from pubertal age multilevel models.

	Mean trajectory (95% CI) of height-adjusted fat mass		**Mean difference in height-adjusted fat mass (95% CI)** **per year later age at peak height velocity**	
Females	Before puberty (% /y)^*^	17.58 (14.26, 20.91)	Before puberty (% difference /y)^‡^	−1.06 (−1.81, −0.31)
	Fat mass at puberty (kg)^†^	11.19 (10.09, 12.30)	Fat mass at puberty (% difference)^§^	−8.62 (−10.60, −6.63)
	After puberty (%/y)^*^	10.57 (8.99, 12.14)	After puberty (% difference /y)^‡^	1.36 (1.03, 1.70)

Males	Up-to 3 years before puberty (% /y)^*^	22.50 (14.60, 30.41)	Up-to 3 years before puberty (% difference/y)^‡^	−5.87 (−7.22, −4.53)
	From 3 years before to puberty (% /y)^*^	−0.12 (−3.52, 3.28)	From 3 years before to puberty (% difference/y )^‡^	3.47 (2.58, 4.37)
	Fat mass at puberty (kg)^†^	8.05 (6.87, 9.22)	Fat mass at puberty (% difference)^§^	−2.31 (−5.16, 0.54)
	After puberty (% /y )^*^	4.65 (1.62, 7.69)	After puberty (% difference /y)^‡^	−0.79 (−1.41, −0.18)

Association of age at peak height velocity (per year later) with change in fat mass before puberty, fat mass at puberty and change in fat mass after puberty in females and males separately. Mean trajectory is centred on the sex-specific mean of age at peak height velocity for each sex (age ∼11.7 for females and age ∼13.6 for males). The difference in fat mass per year of age at peak height velocity is back-transformed from the log scale for ease of interpretation and is a ratio of geometric means, expressed as a percentage difference. Models are adjusted for birth weight, gestational age, maternal education, parity, maternal smoking during pregnancy, maternal age, maternal pre-pregnancy BMI, household social class, marital status, partner education and breastfeeding.

* Percentage change per year in height-adjusted fat mass.

† Mean height-adjusted fat mass at puberty.

‡ Percentage difference in change per year per year later age at peak height velocity.

§ Percentage difference in fat mass at puberty per year later age at peak height velocity.

CI, confidence interval.

### Puberty timing and adiposity change from models by pubertal age

**Figure 2.**
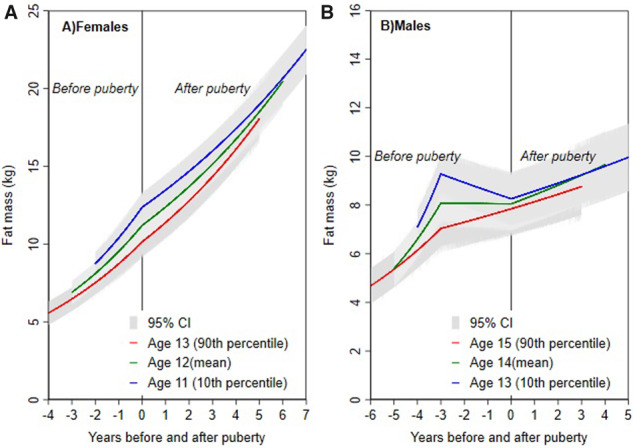
**Mean trajectories of height-adjusted fat mass in females and males from 9 to 18 years for the 10th, median and 90th sex-specific percentiles of age at peak height velocity from multilevel models based on pubertal age.** Ages presented are rounded for ease of interpretation. Exact ages are 12.9, 11.7 and 10.7 years for females and 14.7, 13.6 and 12.5 years for males. Age at peak height velocity is normally distributed and median is equal to mean. Models are adjusted for birth weight, gestational age, maternal education, parity, maternal smoking during pregnancy, maternal age, maternal pre-pregnancy BMI, household social class, marital status, partner education and breastfeeding.

Among females, a 1-year later aPHV was associated with a slower gain in fat mass before puberty, a lower fat mass at puberty and faster gain in fat mass after puberty. In males, up to 3 years before puberty, a 1-year later aPHV was associated with slower gains in fat mass, whereas from 3 years before puberty to pubertal onset, a 1-year later aPHV was associated with faster gain in fat mass. At puberty onset, males with a 1-year later aPHV had lower fat mass, albeit with confidence intervals spanning the null value. After puberty, later aPHV was associated with slower gain in fat mass. Mean-adjusted trajectories of fat mass from 9 to 18 years for the 10th (age 11 in females and 13 males), 50th (age 12 in females and 14 in males) and 90th (age 13 in females and 15 in males) sex-specific percentiles of aPHV by pubertal age are presented in Fig. 2.

Unadjusted and confounder-adjusted results were similar for each analysis (Supplementary Tables SVII and SVIII), regardless of whether unadjusted analyses were performed on N = 5693 participants with data on aPHV and at least one measure of fat mass from 9 to 18 years or on the sample included in our main analysis with complete confounder data too (N = 4176).

### Sensitivity analyses

Estimates of sex-specific associations of aPHV with observed fat mass data at age 9 and 18 years were similar to those obtained from both types of multilevel models (by chronological age and time before and after puberty onset) (Supplementary Table SIX). Results were not appreciably different when analyses were restricted to participants with at least one measure of fat mass before and one measure after aPHV (Supplementary Figs S2 and S3). Results for females were also similar when using self-reported age at menarche rather than aPHV (Supplementary Fig. S4). Our analyses were also not appreciably different when restricted to White ALSPAC participants only (Supplementary Figs S5 and S6).

## Discussion

This study aimed to better understand the nature of puberty timing and adiposity change by examining associations of an objective height-based measure of puberty timing (aPHV) with change in DXA-measured total body fat mass repeatedly measured throughout childhood and adolescence. In females, our findings suggest that earlier puberty timing is more likely to be the result of adiposity gain in childhood than a cause of adiposity gain in adulthood. In males, findings suggest that childhood adiposity may also contribute to early puberty timing and that differences in fat mass after puberty are driven partially by tracking of adiposity from early childhood but also greater gains in post-pubertal adiposity in males early to puberty. Taken together, the findings suggest that reducing levels of childhood adiposity may help to prevent earlier puberty, adult adiposity and its adverse health and social outcomes.

### Comparison with other studies

Our findings in females are consistent with several previous studies showing inverse associations of pre-pubertal BMI and puberty timing (Wang, 2002; Buyken *et al.*, 2008; Kaplowitz, 2008; Silventoinen *et al.*, 2008). For example, in a Swedish study (N = 3650) which used growth data from age 7 to 18 years to estimate aPHV, faster rates of gain in BMI from 2 to 8 years were associated with earlier puberty in females (He and Karlberg, 2001). In a Danish study (N = 156 835), BMI at 7 years was associated with earlier puberty in females, based on onset of growth spurt and aPHV (Aksglaede *et al.*, 2009). These findings are also comparable to a study from the Cardiovascular Risk in Young Finns study (N = 794) which concluded that greater childhood BMI contributed to earlier age at menarche and because of tracking, to greater adult BMI (Kivimèki *et al.*, 2008). Our results are also similar to findings from a recent MR in our cohort which showed that associations of age at menarche with adulthood BMI result from tracking of childhood BMI (Bell *et al.*, 2018). Our findings showed greater gains in fat mass after puberty among females with an older age at puberty which to our knowledge has not been demonstrated previously due to a lack of studies with repeated measures of pre- and post-pubertal fat mass. Though it is difficult to understand the precise reason for this, one possibility includes catch up in fat mass accrual after puberty among females with an older age at puberty due to slower rates of gain in fat mass prior to puberty.

Our results in males are consistent with most (He and Karlberg, 2001; Sandhu *et al.*, 2006; Buyken *et al.*, 2008; Silventoinen *et al.*, 2008; Aksglaede *et al.*, 2009) but not all (Wang, 2002; Lee *et al.*, 2010) previous studies that found that greater adiposity in early childhood is associated with early puberty. The Christ’s Hospital Cohort (N = 1520) showed that males with higher childhood BMI before puberty had earlier aPHV (Sandhu *et al.*, 2006) while in the aforementioned Swedish study, change in BMI from 2 to 8 years was also associated with earlier puberty (He and Karlberg, 2001). Similarly, a Swedish study of 99 monozygotic and 76 dizygotic twins found that early childhood BMI was associated with earlier puberty in males (Silventoinen *et al.*, 2008). One known exception to this is a US study of 401 males which showed that faster gains in BMI from 2 to 11.5 years were associated with later puberty onset in males, based on Tanner staging as assessed by paediatric endocrinologists (Lee *et al.*, 2010). Our findings of greater gains in fat mass up to 3 years before puberty followed by slower gains in fat mass in the period directly before puberty among males early to puberty build on and partially consolidate these inconsistent findings to date in males. Increasing body fat is thought to play a critical role in switching on adrenal androgen secretion leading to the initiation of puberty; this may explain steeper rises in fat mass in males earlier to puberty, in the period up to 3 years before puberty (Kaplowitz, 2008). Once the underlying process of puberty is initiated in males, fat mass decreases and this decrease is steeper in males with a younger age at puberty. These decreases in fat mass in males prior to puberty may be linked to rising testosterone levels in males (Kaplowitz, 2008). aPHV has also been shown to be a marker of more advanced puberty stages in males than in females (Tanner genetalia stages 4 and 5 in males compared with Tanner breast stages 2 and 3 in females) (Granados *et al.*, 2015). These differences may contribute to the different associations between fat mass change and aPHV in females and males as well as to the contrasting associations of aPHV with fat mass change up to 3 years before puberty and then between 3 years before puberty and aPHV.

### Strengths and limitations

The main strengths of our study include the use of an objective measure of puberty timing (aPHV) based on prospective, repeated measures of height from age 5 to 20 years which is a more accurate marker than measures that have been frequently used in previous studies such as age of voice breaking or Tanner staging. We also used repeated measures of adiposity from before to after puberty onset which were directly measured using DXA scans and have not been available in previous studies. Limitations include the lack of measures of fat mass before 9 years and the availability of few measures around puberty, which limit our ability to detect subtle and/or acute changes in fat mass around puberty*.* We aimed to minimise potential selection bias by including all participants with at least one measure of height from 5 to 20 years to estimate aPHV and all participants with at least one measure of fat mass from age 9 to 18 years. In addition, results from analyses with and without selection on complete confounder data were highly similar, indicating a low likelihood of selection driven by missing confounder data. However, while these approaches have minimised selection bias here, selection bias cannot be entirely ruled out. Furthermore, the vast majority of our cohort were of White ethnicity and were socially advantaged. Thus, while we were able to perform a sensitivity analysis restricted to White-only participants, a key limitation of our study is the generalisability of the findings to less advantaged populations and non-White ethnicities.

## Conclusion

Among females, earlier puberty timing is likely to be more of a result of adiposity gain in childhood than a cause of adiposity gain in adulthood. In males, differences in fat mass after puberty are driven partially by tracking of adiposity from early childhood but also by greater gains in post-pubertal adiposity in males earlier to puberty. Interventions aimed at reducing levels of childhood adiposity may help to prevent earlier puberty, adult adiposity and their adverse health outcomes in both females and males. In contrast, interventions aimed at prevention of early puberty without prevention of childhood adiposity would have little downstream benefits for prevention of adult adiposity and cardiovascular risk.

## Supplementary Material

deaa213_Supplementary_Data

deaa213_Supplementary_Figure_S1

deaa213_Supplementary_Figure_S2

deaa213_Supplementary_Figure_S3

deaa213_Supplementary_Figure_S4

deaa213_Supplementary_Figure_S5

deaa213_Supplementary_Figure_S6

deaa213_Supplementary_Table_SI

deaa213_Supplementary_Table_SII

deaa213_Supplementary_Table_SIII

deaa213_Supplementary_Table_SIV

deaa213_Supplementary_Table_SV

deaa213_Supplementary_Table_SVI

deaa213_Supplementary_Table_SVII

deaa213_Supplementary_Table_SVII

deaa213_Supplementary_Table_SIX

## Supplementary materials and methods

### Details of deriving age at peak height velocity

#### Height measures included in the analysis

Height data from questionnaires and health records were excluded. Only data measured at clinic assessments carried out after age 5 years were included in the analysis. A Child in Focus (CIF) clinic measured height using a Leicester Height Measure on a 10% sub-sample of participants measured at 5 years. From 7 years onwards, standing height was measured to the last complete mm using the Harpenden Stadiometer at clinics carried out at ages 7, 9, 10, 11, 13, 14, 15 and 18 years. Data were further restricted to only include individuals with at least one measurement of height in each of the age ranges, 5 to <10, 10 to <15 and 15 to 20 years. The final dataset for analysis included 20 849 height measurements for 2688 boys and 24 216 measurements for 3019 girls. The number of height measures for females and males is shown in Table I. Further details of how age at peak height velocity (aPHV) was derived are described elsewhere (Frysz *et al.*, 2018).

#### Analysis deriving age aPHV

Available height measures were analysed for females and males separately using Superimposition by Translation and Rotation (SITAR) growth curve analysis with five degrees of freedom (Cole *et al.*, 2010). This method is a validated method of deriving aPHV and is described elsewhere in detail (Cole *et al.*, 2010; Simpkin *et al.*, 2017). aPHV was defined as the age when the first derivative of the mean curve, plotted as height versus age, was maximal. After fitting the initial model, the data were checked and points with velocity exceeding four standard deviations (SDs) and standardised residuals exceeding three in absolute value were removed. The model explained 98.5% of variance in males and 98.7% in females. Mean growth curve and velocity plots for females and males are shown in Fig. 1.

### Measurement of confounders included in analysis

Birthweight was extracted from medical records. Gestational age at birth was estimated from clinical records. A questionnaire at 32 weeks of gestation asked mothers to report their educational attainment, which was categorised as below O-Level (Ordinary Level; exams taken in different subjects usually at age 15–16 at the completion of legally required school attendance, equivalent to today’s UK General Certificate of Secondary Education), O-Level only, A-Level (Advanced-Level; exams taken in different subjects usually at age 18), or university degree or above. Parity was defined as the number of previous pregnancies that had resulted in a live- or still-born infant collected at 18 weeks of gestation. Smoking in the first trimester of pregnancy was self-reported by mothers at 18 weeks of gestation. Maternal age was reported in the mother’s antenatal questionnaires. Maternal height and weight were self-reported from the questionnaire administered at 12 weeks of gestation; these were used to calculate maternal BMI. Household social class was measured as the highest of the mother’s or her partner’s occupational social class using data on job title and details of occupation collected about the mother and her partner from the mother’s questionnaire at 32 weeks of gestation. Social class was derived using the standard occupational classification (SOC) codes developed by the United Kingdom Office of Population Census and Surveys and classified as I professional, II managerial and technical, IIINM non-manual, IIIM manual and IV&V part skilled occupations and unskilled occupations. Marital status was obtained from antenatal questionnaires and classified as never married, widowed, divorced, separated, first marriage, marriage two or three. A questionnaire at 32 weeks of gestation asked partners to report their educational attainment, which was categorised as below O-Level (Ordinary Level; exams taken in different subjects usually at age 15–16 years at the completion of legally required school attendance, equivalent to today’s UK General Certificate of Secondary Education), O-Level only, A-Level (Advanced-Level; exams taken in different subjects usually at age 18), or university degree or above. Breastfeeding information used here was collected via questionnaires administered at 4 weeks, 6 and 15 months and categorised as having ever vs. never breastfed.

### Details of model selection

Fat mass was measured on five occasions between 9 and 18 years. Values of fat mass four SDs greater than or less than the mean were excluded from the analysis. Fat mass was log-transformed due to skewness of the data. We included all participants with at least one measure of fat mass in each multilevel model, under a missing at random, to minimise selection bias. The observations of participants that reported being pregnant at the 18-year clinic were excluded from the multilevel models at that time point only. Trajectories were modelled separately for females and males to allow each sex to have different variance-covariance matrices. Models were adjusted for a time- and sex-varying height covariate which was included as a fixed effect, as described elsewhere in detail (O'Keeffe *et al.*, 2019a,b).

We modelled sex-specific change over time in fat mass according to chronological age and pubertal age to better understand the association of aPHV with change in fat mass during childhood and adolescence. In both models, linear splines were used to examine change in fat mass (O'Keeffe *et al.*, 2018a,b).

#### Models based on chronological age

A chronological age model was developed previously and is described elsewhere in detail (O'Keeffe *et al.*, 2018a,b, 2019a,b). Thus, for this analysis, we re-examined the fit of this model according to sex-specific quartiles of pubertal age to ensure that model fit was similar across each of the quartiles of pubertal age and that modifications to this model (including different periods of change or fewer or greater spline periods were not required) for different pubertal age groups. We found that this model had good model fit across sex-specific quartiles of pubertal age. In brief, age in years was centred at 9 years. Knots were placed at 13 and 15 years resulting in three periods of change; from 9 to <13, 13 to <15 and 15 to 18 years. As each sex was modelled separately, the models for males and females took the form of: log fat mass_*ij*_ = *β*_0_ + *u*_0__*j*_ + (*β*_1_+ *u*_1__*j*_) s_*ij*__1_ + (*β*_2_+ *u*_2__*j*__)_ s_*ij*__2_ + (*β*_3_ + *u*_3__*j*__)_ s_*ij*__3_ + *β*_4_ (age and sex adjusted height covariate)_*ij*_ + *e_ij_* where for person *j* at measurement occasion *i*; *β*_0_ represents the fixed effect coefficient for the average intercept, *β*_1_ to *β*_3_ represent fixed effect coefficients for the average linear slopes of each linear spline (s), *β*_4_ represents the fixed effect coefficient for the average difference in measurements between individuals of different heights, *u*_0__*j*_ to *u*_3__*j*_ indicate person-specific random effects for the intercept and slopes, respectively, and *e_ij_* represents the occasion-specific residuals or measurement error which was allowed to vary with age.

#### Models based on pubertal age

Models examining change in fat mass according to pubertal age were modelled *de novo* for this paper. We examined observed data at each age by sex to examine whether the shape of change over time was similar or different between quartiles of pubertal age. We found that the shape of change over time was similar across quartiles of pubertal age in each sex, but that change over time differed for females and males. Therefore, based on the observed data, we examined the fit of a model with two periods of change (pre- and post-puberty) and three periods of change (up to 3 years before puberty, from 3 years before puberty to pubertal onset and from puberty to the end of follow-up) in females and males separately. The model with the best fit in females across each quartile of pubertal age was a two-spline model allowing for a single pre-pubertal change period and a single post-pubertal change period. The model with the best fit in males was a three-spline model allowing for two pre-pubertal change periods and one post-pubertal period of change.

The model for females took the form of log fat mass_*ij*_ = *β*_0_ + *u*_0__*j*_ + (*β*_1_+ *u*_1__*j*_) s_*ij*__1_ + (*β*_2_+ *u*_2__*j*__)_ s_*ij*__2_ + β_3_ (age and sex adjusted height covariate)_*ij*_ + *e_ij_* where for person *j* at measurement occasion *i*; *β*_0_ represents the fixed effect coefficient for the average intercept, *β*_1_ represents the fixed effect coefficients for the average linear slope (s) before puberty, *β*_2_ represents the fixed effect coefficient for the average linear slope (s) after puberty, *β*_3_ represents the fixed effect coefficient for the average difference in measurements between individuals of different heights, *u*_0j_ to *u*_2j_ indicate person-specific random effects for the intercept and slopes, respectively, and *e_ij_* represents the occasion-specific residuals or measurement error which was allowed to vary with age.

The model for males took the form of: log fat mass_*ij*_ = *β*_0_ + *u*_0__*j*_ + (*β*_1_+ *u*_1__*j*_) s_*ij*__1_ + (*β*_2_+ *u*_2__*j*__)_ s_*ij*__2_ + (*β*_3_ + *u*_3__*j*__)_ s_*ij*__3_ + *β*_4_ (age and sex adjusted height covariate)_*ij*_ + *e_ij_* where for person *j* at measurement occasion *i*; *β*_0_ represents the fixed effect coefficient for the average intercept, *β*_1_ represents the fixed effect coefficients for the average linear slope (s) from the first available measure to 3 years before puberty (s), *β*_2_ represents the fixed effect coefficient for the average linear slope (s) from three years before puberty to pubertal onset, *β*_3_ represents the fixed effect coefficient for the average linear slope (s) from pubertal onset to the end of follow-up, *β*_4_ represents the fixed effect coefficient for the average difference in measurements between individuals of different heights, *u*_0__*j*_ to u_3j_ indicate person-specific random effects for the intercept and slopes, respectively, and *e_ij_* represents the occasion-specific residuals or measurement error which was allowed to vary with age.

#### Variance-covariance matrices for the above models

We put no constraints on the variance/covariance matrix of person-specific random effects for all models described above. In each model, we assumed that there was no correlation between the occasion-level random effects (occasion-specific residuals described above). Thus, the occasion-level variance/covariance matrix had all off-diagonal terms set to zero.
