# Supplementary material for: Puberty timing and adiposity change across childhood and adolescence: disentangling cause and consequence
Source: Hum Reprod. 2020 Nov 26;35(12):2784–92. doi: 10.1093/humrep/deaa213 (PMC7744159; doi:10.1093/humrep/deaa213)
Supplement: deaa213_Supplementary_Data [file deaa213_supplementary_data.pdf]

Human Reproduction, pp. 1–1, 2020
doi:10.1093/humrep/deaa213

human reproduction SUPPLEMENTARY DATA

Supplementary materials and methods

Details of deriving age at peak height velocity

Height measures included in the analysis

Height data from questionnaires and health records were excluded. Only data measured at clinic assessments carried out after age 5 years were included in the analysis. A Child in Focus (CIF) clinic measured height using a Leicester Height Measure on a 10% sub-sample of participants measured at 5 years. From 7 years onwards, standing height was measured to the last complete mm using the Harpenden Stadiometer at clinics carried out at ages 7, 9, 10, 11, 13, 14, 15 and 18 years. Data were further restricted to only include individuals with at least one measurement of height in each of the age ranges, 5 to <10, 10 to <15 and 15 to 20 years. The final dataset for analysis included 20 849 height measurements for 2688 boys and 24 216 measurements for 3019 girls. The number of height measures for females and males is shown in Table I. Further details of how age at peak height velocity (aPHV) was derived are described elsewhere (Frysz *et al.*, 2018).

Analysis deriving age aPHV

Available height measures were analysed for females and males separately using Superimposition by Translation and Rotation (SITAR) growth curve analysis with five degrees of freedom (Cole *et al.*, 2010). This method is a validated method of deriving aPHV and is described elsewhere in detail (Cole *et al.*, 2010; Simpkin *et al.*, 2017). aPHV was defined as the age when the first derivative of the mean curve, plotted as height versus age, was maximal. After fitting the initial model, the data were checked and points with velocity exceeding four standard deviations (SDs) and standardised residuals exceeding three in absolute value were removed. The model explained 98.5% of variance in males and 98.7% in females. Mean growth curve and velocity plots for females and males are shown in Fig. 1.

Measurement of confounders included in analysis

Birthweight was extracted from medical records. Gestational age at birth was estimated from clinical records. A questionnaire at 32 weeks of gestation asked mothers to report their educational attainment, which was categorised as below O-Level (Ordinary Level; exams taken in different subjects usually at age 15–16 at the completion of legally required school attendance, equivalent to today's UK General Certificate of Secondary Education), O-Level only, A-Level (Advanced-Level; exams taken in different subjects usually at age 18), or university degree or above. Parity was defined as the number of previous pregnancies that had resulted in a live- or still-born infant collected at 18 weeks of gestation. Smoking in the first trimester of pregnancy was self-reported by mothers at 18 weeks of gestation. Maternal age was reported in the mother's antenatal questionnaires. Maternal height and weight were self-reported from the questionnaire administered at 12 weeks of gestation; these were used to calculate maternal BMI. Household social class was measured as the highest of the mother's or her partner's occupational social class using data on job title and details of occupation collected about the mother and her partner from the mother's questionnaire at 32 weeks of gestation. Social class was derived using the standard occupational classification (SOC) codes developed by the United Kingdom Office of Population Census and Surveys and classified as I professional, II managerial and technical, IIINM non-manual, IIIM manual and IV&V part skilled occupations and unskilled occupations. Marital status was obtained from antenatal questionnaires and classified as never married, widowed, divorced, separated, first marriage, marriage two or three. A questionnaire at 32 weeks of gestation asked partners to report their educational attainment, which was categorised as below O-Level (Ordinary Level; exams taken in different subjects usually at age 15–16 years at the completion of legally required school attendance, equivalent to today's UK General Certificate of Secondary Education), O-Level only, A-Level (Advanced-Level; exams taken in different subjects usually at age 18), or university degree or above. Breastfeeding information used here was collected via questionnaires administered at 4 weeks, 6 and 15 months and categorised as having ever vs. never breastfed.

Details of model selection

Fat mass was measured on five occasions between 9 and 18 years. Values of fat mass four SDs greater than or less than the mean were excluded from the analysis. Fat mass was log-transformed due to skewness of the data. We included all participants with at least one measure of fat mass in each multilevel model, under a missing at random, to minimise selection bias. The observations of participants that reported being pregnant at the 18-year clinic were excluded from the multilevel models at that time point only. Trajectories were modelled separately for females and males to allow each sex to have different variance-covariance matrices. Models were adjusted for a time- and sex-varying height covariate which was included as a fixed effect, as described elsewhere in detail (O'Keeffe *et al.*, 2019a,b).

We modelled sex-specific change over time in fat mass according to chronological age and pubertal age to better understand the association of aPHV with change in fat mass during childhood and adolescence. In both models, linear splines were used to examine change in fat mass (O'Keeffe *et al.*, 2018a,b).

Models based on chronological age

A chronological age model was developed previously and is described elsewhere in detail (O'Keeffe *et al.*, 2018a,b, 2019a,b). Thus, for this analysis, we re-examined the fit of this model according to sex-specific quartiles of pubertal age to ensure that model fit was similar across each of the quartiles of pubertal age and that modifications to this model (including different periods of change or fewer or greater spline periods were not required) for different pubertal age groups. We found that this model had good model fit across sex-specific quartiles

of pubertal age. In brief, age in years was centred at 9 years. Knots were placed at 13 and 15 years resulting in three periods of change; from 9 to <13, 13 to <15 and 15 to 18 years. As each sex was modelled separately, the models for males and females took the form of: log fat mass$_{ij}$ = $\beta_0 + u_{0j} + (\beta_1 + u_{1j})\ s_{ij1} + (\beta_2 + u_{2j})\ s_{ij2} + (\beta_3 + u_{3j})\ s_{ij3} + \beta_4$ (age and sex adjusted height covariate)$_{ij}$ + e_{ij} where for person j at measurement occasion i; β_0 represents the fixed effect coefficient for the average intercept, β_1 to β_3 represent fixed effect coefficients for the average linear slopes of each linear spline (s), β_4 represents the fixed effect coefficient for the average difference in measurements between individuals of different heights, u_{0j} to u_{3j} indicate person-specific random effects for the intercept and slopes, respectively, and e_{ij} represents the occasion-specific residuals or measurement error which was allowed to vary with age.

Models based on pubertal age

Models examining change in fat mass according to pubertal age were modelled *de novo* for this paper. We examined observed data at each age by sex to examine whether the shape of change over time was similar or different between quartiles of pubertal age. We found that the shape of change over time was similar across quartiles of pubertal age in each sex, but that change over time differed for females and males. Therefore, based on the observed data, we examined the fit of a model with two periods of change (pre- and post-puberty) and three periods of change (up to 3 years before puberty, from 3 years before puberty to pubertal onset and from puberty to the end of follow-up) in females and males separately. The model with the best fit in females across each quartile of pubertal age was a two-spline model allowing for a single pre-pubertal change period and a single post-pubertal change period. The model with the best fit in males was a three-spline model allowing for two pre-pubertal change periods and one post-pubertal period of change.

The model for females took the form of log fat mass$_{ij}$ = $\beta_0 + u_{0j} + (\beta_1 + u_{1j})\ s_{ij1} + (\beta_2 + u_{2j})\ s_{ij2} + \beta_3$ (age and sex adjusted height covariate)$_{ij}$ + e_{ij} where for person j at measurement occasion i; β_0 represents the fixed effect coefficient for the average intercept, β_1 represents the fixed effect coefficients for the average linear slope (s) before puberty, β_2 represents the fixed effect coefficient for the average linear slope (s) after puberty, β_3 represents the fixed effect coefficient for the average difference in measurements between individuals of different heights, u_{0j} to u_{2j} indicate person-specific random effects for the intercept and slopes, respectively, and e_{ij} represents the occasion-specific residuals or measurement error which was allowed to vary with age.

The model for males took the form of: log fat mass$_{ij}$ = $\beta_0 + u_{0j} + (\beta_1 + u_{1j})\ s_{ij1} + (\beta_2 + u_{2j})\ s_{ij2} + (\beta_3 + u_{3j})\ s_{ij3} + \beta_4$ (age and sex adjusted height covariate)$_{ij}$ + e_{ij} where for person j at measurement occasion i; β_0 represents the fixed effect coefficient for the average intercept, β_1 represents the fixed effect coefficients for the average linear slope (s) from the first available measure to 3 years before puberty (s), β_2 represents the fixed effect coefficient for the average linear slope (s) from three years before puberty to pubertal onset, β_3 represents the fixed effect coefficient for the average linear slope (s) from pubertal onset to the end of follow-up, β_4 represents the fixed effect coefficient for the average difference in measurements between individuals of different heights, u_{0j} to u_{3j} indicate person-specific random effects for the intercept and slopes, respectively, and e_{ij} represents the occasion-specific residuals or measurement error which was allowed to vary with age.

Variance-covariance matrices for the above models

We put no constraints on the variance/covariance matrix of person-specific random effects for all models described above. In each model, we assumed that there was no correlation between the occasion-level random effects (occasion-specific residuals described above). Thus, the occasion-level variance/covariance matrix had all off-diagonal terms set to zero.
