# Supplementary material for: Puberty timing and adiposity change across childhood and adolescence: disentangling cause and consequence
Source: Hum Reprod. 2020 Nov 26;35(12):2784–92. doi: 10.1093/humrep/deaa213 (PMC7744159; doi:10.1093/humrep/deaa213)
Supplement: deaa213_Supplementary_Figure_S1 [file deaa213_supplementary_figure_s1.pdf]

Human Reproduction, pp. 1–1, 2020
doi:10.1093/humrep/deaa213

human reproduction

SUPPLEMENTARY DATA

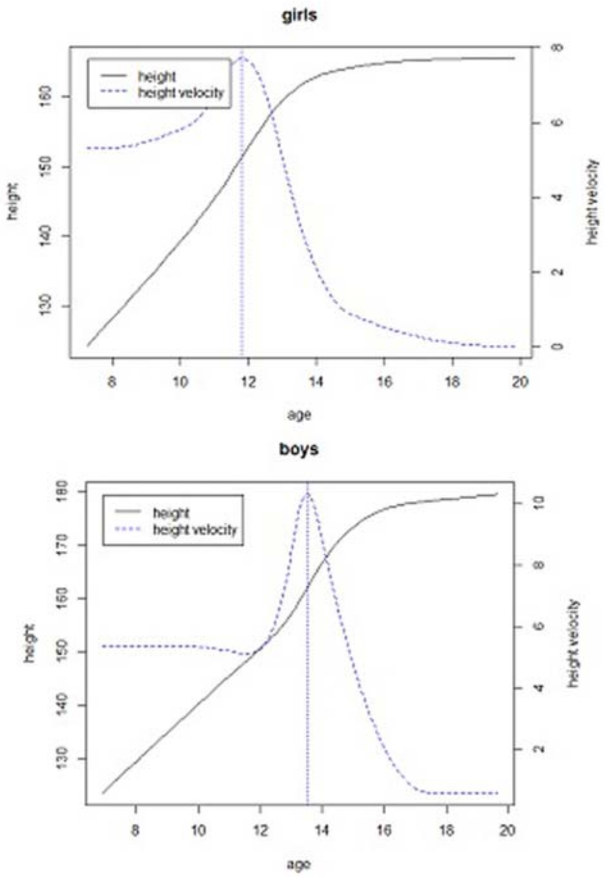

Supplementary Figure S1. Mean growth curve (black line) and velocity (blue dashed line) estimated by Superimposition by Translation and Rotation (SITAR) for females and males. Vertical dotted line represents age at peak height velocity.
