# Supplementary material for: Puberty timing and adiposity change across childhood and adolescence: disentangling cause and consequence
Source: Hum Reprod. 2020 Nov 26;35(12):2784–92. doi: 10.1093/humrep/deaa213 (PMC7744159; doi:10.1093/humrep/deaa213)
Supplement: deaa213_Supplementary_Figure_S6 [file deaa213_supplementary_figure_s6.pdf]

Human Reproduction, pp. 1–1, 2020
doi:10.1093/humrep/deaa213

human reproduction

SUPPLEMENTARY DATA

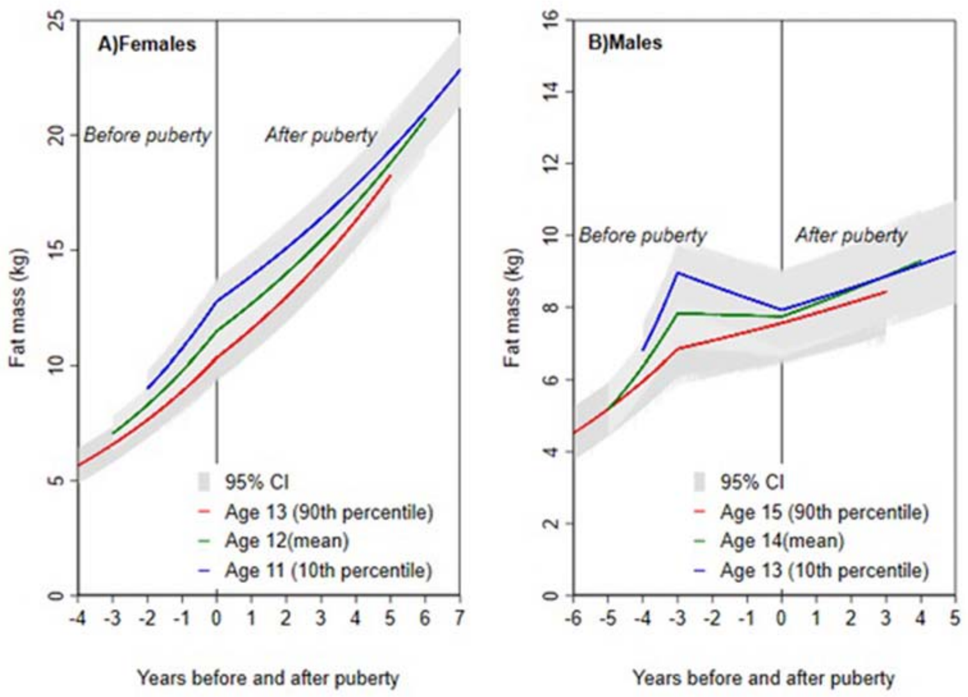

Supplementary Figure S6. Mean trajectories of height-adjusted fat mass in females and males from 9 to 18 years for the 10th, median and 90th sex-specific percentiles of age at peak height velocity from multilevel models based on pubertal age, excluding participants of non-White ethnicity. Ages presented are rounded for ease of interpretation. Exact ages are 12.9, 11.7 and 10.7 years for females and 14.7, 13.6 and 12.5 years for males. Age at peak height velocity is normally distributed and median is equal to mean. Models are adjusted for birth weight, gestational age, maternal education, parity, maternal smoking during pregnancy, maternal age, maternal pre-pregnancy BMI, household social class, marital status, partner education and breastfeeding.
