# Supplementary material for: Puberty timing and adiposity change across childhood and adolescence: disentangling cause and consequence
Source: Hum Reprod. 2020 Nov 26;35(12):2784–92. doi: 10.1093/humrep/deaa213 (PMC7744159; doi:10.1093/humrep/deaa213)
Supplement: deaa213_Supplementary_Table_SI [file deaa213_supplementary_table_si.pdf]

Human Reproduction, pp. 1–1, 2020
doi:10.1093/humrep/deaa213

human reproduction

SUPPLEMENTARY DATA

Supplementary Table SI **Details of height measures available for deriving age at peak height velocity.**

Males			Females		
**No. of height measurements per individual**	**Number of individuals**	**%**	**No. of height measurements per individual**	**Number of individuals**	**%**
1	8	0.3	N/A		
2	24	0.89	<5*	18	0.6
3	26	0.97	<5*	31	1.03
4	70	2.6	<5*	60	1.99
5	120	4.5	5	100	3.31
6	219	8.2	6	192	6.36
7	432	16.1	7	355	11.8
8	638	23.7	8	826	27.4
9	1117	41.6	9	1268	42
10	34	1.26	10	169	5.6
Total	2688	100%	Total	3019	100%

*Exact numbers not shown due to potential for disclosure.
