# Supplementary material for: Puberty timing and adiposity change across childhood and adolescence: disentangling cause and consequence
Source: Hum Reprod. 2020 Nov 26;35(12):2784–92. doi: 10.1093/humrep/deaa213 (PMC7744159; doi:10.1093/humrep/deaa213)
Supplement: deaa213_Supplementary_Table_SII [file deaa213_supplementary_table_sii.pdf]

Human Reproduction, pp. 1–2, 2020
doi:10.1093/humrep/deaa213

human reproduction

SUPPLEMENTARY DATA

Supplementary Table SII **Distributions/proportions for confounders inclusive of missing data by fourths of age at peak height velocity in N = 5704 participants with data on age at peak height velocity.**

	Quartile 1 n (%)	Quartile 2 n (%)	Quartile 3 n (%)	Quartile 4 n (%)
Maternal marital status				
Never married	175 (12.3)	188 (13.2)	170 (11.9)	186 (13.0)
Widowed	<5*	<5*	<5*	<5*
Divorced	53 (3.7)	44 (3.1)	49 (3.4)	45 (3.2)
Separated	16 (1.1)	16 (1.1)	11 (0.8)	10 (0.7)
1st Marriage	1014 (71.1)	1009 (70.8)	1006 (70.5)	1006 (70.5)
Marriage 2 or 3	79 (5.5)	77 (5.4)	92 (6.5)	84 (5.9)
Missing	88 (6.2)	90 (6.3)	96 (6.7)	94 (6.6)
Household social class				
Professional	208 (14.6)	209 (14.7)	239 (16.8)	237 (16.6)
Managerial & Technical	588 (41.2)	567 (39.8)	582 (40.8)	592 (41.5)
Non-Manual	303 (21.2)	329 (23.1)	292 (20.5)	281 (19.7)
Manual	117 (8.2)	109 (7.6)	126 (8.8)	103 (7.2)
Part Skilled & Unskilled	60 (4.2)	52 (3.6)	36 (2.5)	44 (3.1)
Missing	150 (10.5)	160 (11.2)	151 (10.6)	169 (11.9)
Maternal education				
Less than O level	271 (19.0)	234 (16.4)	234 (16.4)	248 (17.4)
O level	458 (32.1)	485 (34.0)	452 (31.7)	441 (30.9)
A level	360 (25.2)	364 (25.5)	395 (27.7)	384 (26.9)
Degree or above	236 (16.5)	235 (16.5)	238 (16.7)	250 (17.5)
Missing	101 (7.1)	108 (7.6)	107 (7.5)	103 (7.2)
Partners highest educational qualification				
Less than O level	372 (26.1)	313 (21.9)	313 (21.9)	315 (22.1)
O level	274 (19.2)	278 (19.5)	279 (19.6)	286 (20.1)
A level	352 (24.7)	394 (27.6)	363 (25.5)	360 (25.2)
Degree or Above	293 (20.5)	300 (21.0)	332 (23.3)	333 (23.4)
Missing	135 (9.5)	141 (9.9)	139 (9.7)	132 (9.3)
Smoking in pregnancy				
Yes	237 (16.6)	204 (14.3)	199 (14.0)	209 (14.7)
Missing	93 (6.5)	81 (5.7)	92 (6.5)	89 (6.2)
Parity				
0	659 (46.2)	636 (44.6)	661 (46.4)	617 (43.3)
1	449 (31.5)	478 (33.5)	431 (30.2)	470 (33.0)
2	202 (14.2)	213 (14.9)	222 (15.6)	235 (16.5)
Missing	116 (8.1)	99 (6.9)	112 (7.9)	104 (7.3)

(continued)

Supplementary Table SII Continued

	Quartile 1 n (%)	Quartile 2 n (%)	Quartile 3 n (%)	Quartile 4 n (%)
Breastfeeding				
Ever	1048 (73.5)	1048 (73.5)	1089 (76.4)	1048 (73.5)
Never	209 (14.7)	209 (14.7)	164 (11.5)	209 (14.7)
Missing	169 (11.9)	169 (11.9)	173 (12.1)	169 (11.9)
	**Mean (SD)/n (%)**	**Mean (SD)/n (%)**	**Mean (SD)/n (%)**	**Mean (SD)/n (%)**
Gestational age at birth (weeks)	39.5 (1.8)	40 (1.8)	39 (1.9)	39 (2.0)
Missing	66 (4.6)	60 (4.2)	76 (5.3)	63 (4.4)
Birthweight (g)	3345 (512)	3401 (522)	3446 (567)	3487 (587)
Missing	84 (5.9)	78 (5.5)	95 (6.7)	76 (5.3)
Maternal BMI (kg/m^2)	23.1 (3.8)	23 (3.7)	23 (3.7)	23 (3.6)
Missing	195 (13.7)	180 (12.6)	180 (12.6)	182 (12.8)
Maternal age (years)	29.1 (4.4)	29 (4.6)	29 (4.5)	29 (4.6)
Missing	66 (4.6)	60 (4.2)	76 (5.3)	63 (4.4)

N = 5704 represents N with data on age at peak height velocity alone (i.e. not selected based on having data on any other variables included in our analysis). Legend: Quartile 1 = 9–11.6 years; Quartile 2 = 11. 7–12.5 years; Quartile 3 = 12.6–13.5 years; Quartile 4 = 13.6–17.1 years.

*Exact numbers and percentages are not shown due to potential for disclosure.
