# Supplementary material for: Puberty timing and adiposity change across childhood and adolescence: disentangling cause and consequence
Source: Hum Reprod. 2020 Nov 26;35(12):2784–92. doi: 10.1093/humrep/deaa213 (PMC7744159; doi:10.1093/humrep/deaa213)
Supplement: deaa213_Supplementary_Table_SIII [file deaa213_supplementary_table_siii.pdf]

**Human Reproduction, pp. 1–1, 2020**
doi:10.1093/humrep/deaa213

human reproduction

# SUPPLEMENTARY DATA

**Supplementary Table SIII** **Model details for log fat mass trajectories modelled using chronological age, by sex and sex-specific quartiles of pubertal age.**

| | No of contributing individuals | | Assessment of model fit | | | |
|---|---|---|---|---|---|---|
| | Total number of observations | Number of individuals with 1 measure | Mean observed (SD), log fat mass in kg | Mean predicted (SD), log fat mass in kg | Mean difference (observed—predicted), log fat mass in kg | 95% level of agreement between observed and predicted, log fat mass in kg |
| Females | | | | | | |
| Overall | 9565 | 2186 | | | | |
| 1st quartile (9.1–11.2) | 2394 | 555 | 2.78 (0.48) | 2.79 (0.44) | −0.002 | −0.25 to 0.24 |
| 2nd quartile (>11.2–11.7) | 2390 | 549 | 2.65 (0.51) | 2.65 (0.48) | −0.002 | −0.24 to 0.24 |
| 3rd quartile (>11.7–12.3) | 2390 | 549 | 2.56 (0.53) | 2.56 (0.49) | 0.0005 | −0.23 to 0.23 |
| 4th quartile (>12.3–14.6) | 2391 | 533 | 2.39 (0.56) | 2.39 (0.53) | −0.001 | −0.24 to 0.23 |
| Males | | | | | | |
| Overall | 8667 | 1990 | | | | |
| 1st quartile (10.8–13) | 2167 | 501 | 2.29 (0.62) | 2.29 (0.55) | 0.001 | −0.36 to 0.36 |
| 2nd quartile (>13–13.6) | 2169 | 497 | 2.14 (0.61) | 2.14 (0.54) | −0.001 | −0.35 to 0.35 |
| 3rd quartile (>13.6–14.2) | 2168 | 499 | 2.11 (0.62) | 2.11 (0.56) | 0.002 | −0.33 to 0.34 |
| 4th quartile (>14.2–17.1) | 2163 | 493 | 1.96 (0.63) | 1.96 (0.58) | −0.001 | −0.31 to 0.31 |
