# Supplementary material for: Puberty timing and adiposity change across childhood and adolescence: disentangling cause and consequence
Source: Hum Reprod. 2020 Nov 26;35(12):2784–92. doi: 10.1093/humrep/deaa213 (PMC7744159; doi:10.1093/humrep/deaa213)
Supplement: deaa213_Supplementary_Table_SIV [file deaa213_supplementary_table_siv.pdf]

Human Reproduction, pp. 1–1, 2020
doi:10.1093/humrep/deaa213

human reproduction

SUPPLEMENTARY DATA

Supplementary Table SIV **Model details for log fat mass trajectories modelled using pubertal age, by sex and sex-specific quartiles of pubertal age.**

	No of contributing individuals		Assessment of model fit			
	Total number of observations	Number of individuals with 1 measure	Mean observed (SD), log fat mass in kg	Mean predicted (SD), log fat mass in kg	Mean difference (observed——predicted), log fat mass in kg	95% level of agreement between observed and predicted, ln log fat mass in kg
Females						
Overall	9565	2186				
1st quartile (9.1–11.2)	2394	555	2.78 (0.48)	2.78 (0.44)	0.001	−0.24 to 0.24
2nd quartile (>11.2–11.7)	2390	549	2.65 (0.51)	2.65 (0.47)	0.0001	−0.24 to 0.24
3rd quartile (>11.7–12.3)	2390	549	2.56 (0.53)	2.56 (0.49)	0.0001	−0.23 to 0.23
4th quartile (>12.3–14.6)	2391	533	2.39 (0.56)	2.39 (0.53)	−0.001	−0.24 to 0.23
Males	8667	1990				
Overall	2167	501				
1st quartile (10.8–13)	2169	497	2.29 (0.62)	2.29 (0.56)	0.001	−0.34 to 0.34
2nd quartile (>13–13.6)	2168	499	2.14 (0.61)	2.14 (0.55)	−0.001	−0.33 to 0.33
3rd quartile (>13.6–14.2)	2163	493	2.11 (0.62)	2.11 (0.56)	0.001	−0.31 to 0.31
4th quartile (>14.2–17.1)	9565	2186	1.96 (0.63)	1.96 (0.58)	−0.001	−0.29 to 0.29
