# Supplementary material for: Puberty timing and adiposity change across childhood and adolescence: disentangling cause and consequence
Source: Hum Reprod. 2020 Nov 26;35(12):2784–92. doi: 10.1093/humrep/deaa213 (PMC7744159; doi:10.1093/humrep/deaa213)
Supplement: deaa213_Supplementary_Table_SV [file deaa213_supplementary_table_sv.pdf]

Human Reproduction, pp. 1–1, 2020
doi:10.1093/humrep/deaa213

human reproduction

SUPPLEMENTARY DATA

Supplementary Table SV **Results from likelihood ratio test examining linearity of association between age at peak height velocity and log fat mass at each age by sex.**

Age (y)	Females<br>*P*-value comparing models	Males<br>*P*-value comparing models
9	0.12	0.38
11	0.49	0.25
13	0.01	0.19
15	0.01	0.12
18	0.63	0.89

P-value from likelihood ratio test comparing fit of a models regressing pubertal age treated as a continuous exposure on fat mass at each age to models regressing fourths of pubertal age also treated as a continuous exposure on fat mass at each age. $P > 0.05$ indicates the more parsimonious model (pubertal age treated as continuous exposure) is a better fit, suggesting linearity of associations of age at peak height velocity and fat mass. Note that although the *P*-value for females at age 13 and 15 indicated some departure from linearity, associations were still broadly linear. Thus, given the linearity of all other associations in females and males, age at peak height velocity was examined as a continuous exposure in our analyses.
