# Supplementary material for: Puberty timing and adiposity change across childhood and adolescence: disentangling cause and consequence
Source: Hum Reprod. 2020 Nov 26;35(12):2784–92. doi: 10.1093/humrep/deaa213 (PMC7744159; doi:10.1093/humrep/deaa213)
Supplement: deaa213_Supplementary_Table_SVI [file deaa213_supplementary_table_svi.pdf]

Human Reproduction, pp. 1–1, 2020
doi:10.1093/humrep/deaa213

human reproduction

SUPPLEMENTARY DATA

Supplementary Table SVI **Characteristics at birth of the mothers of children included in models compared with those excluded due to missing exposure, outcome or co-variate data.**

	Participants included n = 4176 n (%)	Participants excluded with observed data* n (%)	N participants excluded who had observed data†
Maternal marital status			9388
Never married	465 (11.1)	2134 (22.7)	
Widowed	<5‡	14 (0.1)	
Divorced	135 (3.2)	444 (4.7)	
Separated	35 (0.8)	184 (2.0)	
1st Marriage	3265 (78.2)	5999 (63.9)	
Marriage 2 or 3	272 (6.5)	613 (6.5)	
Household social class			7409
Professional	767 (18.4)	774 (10.4)	
Managerial & Technical	1956 (46.8)	2883 (38.9)	
Non-Manual	975 (23.3)	1973 (26.6)	
Manual	340 (8.1)	1224 (16.5)	
Part Skilled & Unskilled	138 (3.3)	555 (7.5)	
Maternal education			8323
Less than O level	671 (16.1)	3085 (37.1)	
O level	1475 (35.3)	2854 (34.3)	
A level	1223 (29.3)	1581 (19.0)	
Degree or above	807 (19.3)	803 (9.6)	
Partners education			7841
Less than O level	979 (23.4)	3175 (40.5)	
O level	915 (21.9)	1641 (20.9)	
A level	1223 (29.3)	1900 (24.2)	
Degree or Above	1059 (25.4)	1125 (14.3)	
Maternal smoking during pregnancy			9188
Yes	3581 (85.8)	6416 (69.8)	
No	595 (14.2)	2772 (30.2)	
Parity			8953
0	2049 (49.1)	3826 (42.7)	
1	1480 (35.4)	3108 (34.7)	
2	647 (15.5)	2019 (22.6)	
Sex			
Female	2186 (52.3)	4647 (46.6)	9972
Male	1990 (47.7)	5325 (53.4)	
	**Mean (SD)**	**Mean (SD)**	
Child gestational age at birth (weeks)	39.5 (1.7)	39.4 (2.0)	9879
Birthweight (g)	3435.1 (530.6)	3372.2 (571.4)	9679
Maternal BMI (kg/m^2)	22.8 (3.7)	23 (3.9)	7517
Maternal age (years)	29.5 (4.4)	27.4 (5.1)	9911
Mean age at peak height velocity (years)	12. 6 (1.3)	12.6 (1.3)	1533
Mean fat mass at 9y (kg)	8.3 (4.8)	8.8 (5.2)	3527

(continued)

Supplementary Table SVI Continued

	Participants included n = 4176 n (%)	Participants excluded with observed data* n (%)	N participants excluded who had observed data†
Mean fat mass at 11y (kg)	11.4 (6.4)	12.0 (7.0)	3139
Mean fat mass at 13y (kg)	13.4 (7.7)	14.1 (8.2)	2288
Mean fat mass at 15y (kg)	14.9 (8.6)	16.0 (9.4)	1518
Mean fat mass at 18y (kg)	17.6 (9.9)	19.3 (10.7)	1581

*Denominators for excluded participants in this table vary due to missing data for characteristics shown.

†This column shows the total number of participants in the cohort who had observed data for the variable but whom were not included in the analysis due to missing data on other variables required for inclusion, i.e. for marital status 9388 participants were excluded from our analysis due to missing data on some other variable/variables but had observed data on marital status.

‡Exact numbers and percentages not shown due to potential for disclosure.

SD, standard deviation.
