# Supplementary material for: Puberty timing and adiposity change across childhood and adolescence: disentangling cause and consequence
Source: Hum Reprod. 2020 Nov 26;35(12):2784–92. doi: 10.1093/humrep/deaa213 (PMC7744159; doi:10.1093/humrep/deaa213)
Supplement: deaa213_Supplementary_Table_SVII [file deaa213_supplementary_table_svii.pdf]

Human Reproduction, pp. 1–1, 2020
doi:10.1093/humrep/deaa213

human reproduction

SUPPLEMENTARY DATA

Supplementary Table SVII Unadjusted mean trajectory and mean difference in trajectory of height-adjusted fat mass per year later age at peak height velocity, from chronological age multilevel models.

		Mean trajectory (95% CI) of height-adjusted fat mass		Mean difference in height-adjusted fat mass (95% CI)
Females				
Unadjusted analysis on N = 2186 (Main analysis sample)§	Age 9y (kg)*	7.43 (7.23, 7.64)	Age 9y (% difference)†	−21.29 (−23.22, −19.36)
	9 - <13y (% /y)**	16.23 (15.31, 17.14)	9 - <13y (% difference /y)‡	0.52 (0.05, 1.00)
	13 - <15y (%/y)**	11.20 (10.35, 12.06)	13 - <15y (% difference /y)‡	2.71 (1.89, 3.53)
	15 - 18y (% /y)**	6.03 (5.55, 6.52)	15–18y (% difference /y)‡	2.40 (1.85, 2.95)
	Age 18y (kg)*	19.99 (19.63, 20.36)	Age 18y (% difference)†	−8.98 (−10.90, −7.05)
Unadjusted analysis on N = 3006¶	Age 9y (kg)*	7.42 (7.25, 7.60)	Age 9y (% difference)†	−21.63 (−23.31, −19.95)
	9 - <13y (% /y)**	16.56 (15.76, 17.36)	9 - <13y (% difference /y)‡	0.44 (0.02, 0.86)
	13 - <15y (%/y)**	10.96 (10.21, 11.70)	13 - <15y (% difference /y)‡	2.72 (1.99, 3.44)
	15–18y (% /y)**	6.03 (5.61, 6.46)	15–18y (% difference /y)‡	2.50 (2.02, 2.99)
	Age 18y (kg)*	20.11 (19.80, 20.43)	Age 18y (% difference)†	−9.37 (−11.02, −7.72)
Males				
Unadjusted analysis on N = 1990 (Main analysis sample)§	Age 9y (kg)*	5.94 (5.73, 6.15)	Age 9y (% difference)†	−24.04 (−26.18, −21.90)
	9 - <13y (% /y)**	12.13 (10.92, 13.34)	9 - <13y (% difference /y)‡	4.84 (4.23, 5.45)
	13 - <15y (%/y)**	−5.87 (−6.92, −4.82)	13 - <15y (% difference /y)‡	1.39 (0.31, 2.46)
	15–18y (% /y)**	10.11 (9.27, 10.96)	15–18y (% difference /y)‡	−2.72 (−3.56, −1.88)
	Age 18y (kg)*	11.10 (10.77, 11.43)	Age 18y (% difference)†	−13.16 (−16.03, −10.30)
Unadjusted analysis on N = 2677¶	Age 9y (kg)*	6.05 (5.87, 6.24)	Age 9y (% difference)†	−25.28 (−27.11, −23.46)
	9 - <13y (% /y)**	11.95 (10.91, 12.99)	9 - <13y (% difference /y)‡	5.02 (4.49, 5.55)
	13 - <15y (%/y)**	−5.64 (−6.54, −4.74)	13 - <15y (% difference /y)‡	1.13 (0.22, 2.03)
	15–18y (% /y)**	10.14 (9.39, 10.89)	15–18y (% difference /y)‡	−2.64 (−3.37, −1.91)
	Age 18y (kg)*	11.31 (11.02, 11.61)	Age 18y (% difference)†	−14.23 (−16.68, −11.78)

Mean trajectory is centred on the sex-specific mean of age at peak height velocity for each sex (age ~11.7 for females and age ~13.6 for males). The difference in fat mass per year of age at peak height velocity is back-transformed from the log scale for ease of interpretation and is a ratio of geometric means, expressed as a percentage difference.
*Mean height-adjusted fat mass at 9 and 18 years in kilograms.
**Percentage change per year in height-adjusted fat mass.
†Percentage difference in fat mass at 9 and 18 years per year later age at peak height velocity.
‡Percentage difference in change per year per year later age at peak height velocity.
§This sample corresponds to the sample of participants included in our main analysis (Tables II and III in main text); these participants had data on age at peak height velocity, at least one measure of fat mass from 9 to 18 years and all confounders.
¶This sample includes all participants that had a measure of age at peak height velocity and at least one measure of fat mass from 9 to 18 years but whom may not have all had data on confounders as with the main analysis sample.
CI, confidence interval.
