# Supplementary material for: Puberty timing and adiposity change across childhood and adolescence: disentangling cause and consequence
Source: Hum Reprod. 2020 Nov 26;35(12):2784–92. doi: 10.1093/humrep/deaa213 (PMC7744159; doi:10.1093/humrep/deaa213)
Supplement: deaa213_Supplementary_Table_SIX [file deaa213_supplementary_table_six.pdf]

Human Reproduction, pp. 1–1, 2020
doi:10.1093/humrep/deaa213

human reproduction

SUPPLEMENTARY DATA

Supplementary Table SIX Association of pubertal timing with first and last available measure of log fat mass using linear regression compared with predicted differences at 9 and 18 years from multilevel models.

	Association of age at peak height velocity with log fat mass from regression (95% CI)	Association of age at peak height velocity with log fat mass from multilevel model based on pubertal age (95% CI)	Association of age at peak height velocity with log fat mass from multilevel model based on chronological age (95% CI)
Females			
Log fat mass (kg)			
Age 9	0.24 (0.22, 0.27)	0.22 (0.19, 0.25)	0.24 (0.22, 0.26)
Age 18	0.11 (0.09, 0.14)	0.13 (0.10, 0.15)	0.09 (0.07, 0.11)
Males			
Log fat mass (kg)			
Age 9	0.21 (0.18, 0.24)	0.32 (0.28, 0.35)	0.28 (0.25, 0.31)
Age 18	0.13 (0.09, 0.17)	0.10 (0.07, 0.13)	0.14 (0.11, 0.18)
